# Results of screening for retinopathy of prematurity in a large nursery in Kuwait: Incidence and risk factors

**DOI:** 10.4103/0301-4738.62644

**Published:** 2010

**Authors:** Vivek B Wani, Niranjan Kumar, Khalid Sabti, Seemant Raizada, Nabeel Rashwan, Mumtaz M Shukkur, Mohammed Harbi

**Affiliations:** 1 Al Bahar Ophthalmology Center, Kuwait city, Kuwait; 2 Department of Surgery Faculty of Medicine, Kuwait University, Jabriya, Kuwait; 3 Department of Pediatrics, Al Sabah Maternity Hospital, Jabriya, Kuwait; 4 Department of Community Medicine, Faculty of Medicine, Kuwait University, Jabriya, Kuwait

**Keywords:** Incidence, laser photocoagulation, retinopathy of prematurity, risk factors, threshold disease

## Abstract

**Aims:**

The aim of the study was to report the incidence of retinopathy of prematurity (ROP) and severe ROP and identify the risk factors for their development in a large nursery in Kuwait.

**Materials and Methods:**

This was a retrospective, interventional, non-comparative, hospital-based study. Retrospective review of ROP records of premature babies having either birth weight of less than 1501 g or gestational age at birth of 34 weeks or less and born between January 2001 and August 2003.

**Statistical Analysis:**

By univariate and multivariate logistic regression analysis.

**Results:**

Out of the 599 babies studied, 38.9% developed ROP and 7.8% needed treatment for severe ROP. Multivariate analysis showed low birth weight (OR 13.753, 95% CI 3.66-51.54; (*P* < 0.001), gestational age (OR 13.75, 95% CI 3.66-51.54; *P* < 0.001), surfactant (OR 1.72, 95% CI 1.04-2.83; *P* = 0.032) and stay in the intensive care unit for longer than 15 days (OR 2.25, 95% CI 1.05-4.85; *P* = 0.033) to be significant for the development of any ROP. Low birth weight (OR 22.86, 95% CI 3.86-134.82; *P* = 0.001), bacterial sepsis (OR 3.27, 95% CI 1.51-7.05; *P* = 0.002) and need for surfactant (OR 4.41, 95% CI 0.94 -20.56; *P* = 0.059) were found to be the risk factors for severe ROP needing laser treatment.

**Conclusion:**

The incidence of both any ROP and ROP needing treatment are comparable to other studies. Low birth weight is the most important risk factor for both any ROP and severe ROP.

Retinopathy of prematurity (ROP) is a well-established disease of premature babies and has emerged as an important cause of childhood blindness.[1,2] After the cryotherapy for retinopathy of prematurity (CRYO-ROP) study[3] established the benefit of treatment of ROP when it reaches the stage of threshold disease, screening of premature babies for ROP has become an important measure in preventing blindness due to ROP. The reported incidence of acute ROP varies from 9 to 65.8% and that of threshold ROP varies from 0.79 to 27%.[4–9]

The risk factors for the development of ROP and threshold ROP include low birth weight,[6,7,10–12] low gestational age (GA) at birth,[5,6,10–12] oxygen administration or duration of ventilation,[5,6,10,11] sepsis,[6,7,10] number of days of stay in the hospital,[10] intraventricular hemorrhage,[7,13] surfactant therapy[6] and Candidemia.[14]

There are only a few studies from the Arabian Gulf region regarding the incidence of ROP and the risk factors for the development of ROP.[12,15–17] The objective of this study was to report the incidence of any ROP, incidence of severe ROP needing treatment and the risk factors for them in a large nursery in Kuwait.

## Materials and Methods

In a retrospective, interventional, non-comparative, single-center case series study, we reviewed the medical records and ROP charts of premature babies who were born between January 1, 2001 and August 31, 2003 at the neonatal intensive care unit, Al Sabah maternity hospital, Kuwait city, Kuwait. Approval was obtained from the Institutional Ethics Committee for the study. Efforts were made to remain true to the guidelines of the Declaration of Helsinki Principles.

Babies either with a birth weight of less than 1501 g or born at a gestational age of 34 weeks or less were screened for ROP by ophthalmologists if they survived up to 28 days. The first examination was conducted at four weeks of chronological age. Follow-up examination was done every two weeks in the absence of active ROP and weekly or earlier if active ROP was detected. In this study we treated babies when the ROP reached Stage 3 in Zone I or II with 3 clock h of contiguous or 5 clock h of cumulative extraretinal proliferation in the presence of plus disease. We termed this stage as modified threshold disease (MTD) in our study. Babies who did not develop ROP or those who developed acute ROP but did not reach MTD were followed up till the retina became fully vascularized or achieved 50 weeks of gestational age. Dilatation of the pupils was achieved with cyclopentolate 0.5% eye drops instilled three times at 15 min-intervals prior to the screening examination. The findings of indirect ophthalmoscopy were recorded in a ROP datasheet at each visit.

Acute ROP was classified according to the international classification of retinopathy of prematurity (ICROP) study recommendations.[18] Babies were treated by transpupillary diode laser photocoagulation if they reached the stage of MTD or higher. Written informed consent was taken from the parents for laser treatment.

Data were collected for each baby regarding date of birth, sex, single or multiple pregnancy, gestational age at birth, birth weight, surfactant given or not and presence of common problems of prematurity like intraventricular hemorrhage (IVH), bacterial or fungal or combined infection, hyaline membrane disease (HMD) and its severity, necrotizing enterocolitis (NEC) and patent ductus arteriosus (PDA) and its treatment. Presence of intrauterine growth retardation (IUGR) and duration of stay of the infant in the intensive care unit of the neonatology (NICU) department were also noted.

### Statistical analysis

All data was entered into a spreadsheet and analyzed using SPSS for Windows statistical software Version 10.0 (SPSS Inc., Chicago, IL, USA). Univariate and multivariate logistic regression was done to determine the risk factors for the development of any ROP and MTD. A *P* value of 0.05 or less was considered statistically significant. Odds ratio and 95% confidence intervals were calculated for determining the independent risk factors.

## Results

There were 624 babies eligible for screening for ROP. However, 13 babies died before the completion of screening and another 12 babies did not follow up fully for screening examination. After excluding these 25 babies, we identified 599 babies who had completed the examination.

There were 313 (52.3%) male and 286 (47.8%) female babies. The average birth weight of 599 babies was 1270.01 g (range: 490-2150 g, SD 296 g). The average gestational age was 31.1 weeks (range: 24-36weeks, SD ± 2.40 weeks). Three hundred and seventy-four babies (62.4%) were the product of single pregnancy and 225 babies (37.6%) were product of multiple pregnancies. The 225 babies included 116 twins (19.7%), 66 triplets (11.1%), 38 quadruplets (6.3%) and five quintuplets (0.8%).

A significant number of babies had a stormy neonatal course with IVH in 84 (14.0%), proven bacterial sepsis in 121 (20.2%), fungal infection in 27 (4.5%) and mixed fungal and bacterial infection in 12 (2.0%) babies. Nearly 50% of babies (295) had HMD of varying degree. Administration of surfactant was needed in 353 (58.9%). PDA needed administration of indomethacin treatment in 43 babies (7.7%) and surgical ligation in 10 babies (1.67%). IUGR was present in 42 (7.0%) and NEC in 20 babies (3.3%).

Some ROP was detected in 233 babies (38.9%) in at least one eye and the remaining 366 (61.1%) babies did not develop any ROP. Bilateral ROP was detected in 208 (34.7%) and unilateral disease was found in 25 babies (4.2%). The highest stage of ROP reached, in at least one eye in these babies, is shown in Tables 1 and 2.

**Table 1:** Incidence of ROP according to birth weight in grams and stage of highest retinopathy of prematurity reached in at least one eye at the time of decision to treat

Category	No. ROP	Stage 1	Stage 2	Stage 3	MTD*	Total
≤800	3	5	15	2	20	45
801-1000	19	21	24	10	20	94
1001-1250	76	28	17	6	5	132
≥1251	268	40	15	3	2	328
Total	366	94	71	21	47	599

* MTD-Modified threshold disease

The average birth weight of babies developing ROP and MTD was 1077.73 g (range: 490-1900, SD, 279.36) and 874.68 g (range: 490-1635, SD 183.68) respectively. The average GA of babies developing ROP and MTD was 29.66 (range: 24-35, SD 2.52) and 27.74 (range: 24-34, SD 2.07) weeks respectively.

Eighty-six eyes of 47 (7.8%) babies were found to have MTD or worse and were treated by laser ablation. Bilateral disease was seen in 39 (6.5%) babies and unilateral disease was seen in eight (1.3%) babies. Seventy-nine eyes of 43 babies had MTD in Zone II and seven eyes of four babies had MTD in Zone I. Thirteen eyes needed retreatment for skipped areas. All the unilateral cases showed good regression of MTD after laser treatment, but five of 39 cases with bilateral disease showed progression of disease even after laser treatment. One baby, who showed bilateral progression to Stage 4A had spontaneous regression in both eyes. In another baby, Stage 4A in one eye was successfully treated by scleral buckling and the other eye had spontaneous regression of Stage 4A. Two babies showed progression to Stage 5 in both eyes and parents refused surgery. The fifth child progressed to Stage 4B in both eyes and underwent pars plana vitrectomy in both eyes. The child had successful re-attachment of the retina in one eye. In summary, three children (6.4% of treated babies) had unfavorable outcome in at least one eye. Unfavorable structural outcome was defined as total retinal detachment or presence of macular fold.[3] Seventy eyes of 39 infants were treated as per our goal of MTD but 16 eyes of eight patients had progressed to classical threshold or worse. Only one of the 70 (1.4%) eyes treated at MTD had unfavorable structural outcome. This eye had Zone I disease. Out of the 16 eyes treated at classical threshold disease stage, four (25%) eyes had unfavorable structural outcome.

**Table 2:** Incidence of ROP according to gestational age in weeks and stage of highest retinopathy of prematurity reached in at least one eye at the time of decision to treat

Category	No. ROP	Stage 1	Stage 2	Stage 3	MTD*	Total
≤28	14	13	31	13	36	107
29-31	106	38	24	6	7	181
≥32	246	43	16	2	4	311
Total	366	94	71	21	47	599

* MTD-Modified threshold disease

Univariate analysis showed birth weight (*P* < 0.001), GA (*P* < 0.001), presence of IVH (*P* < 0.001), need for surfactant (*P* < 0.001), presence of bacterial infection (*P* < 0.001), presence of fungal infection (*P* < 0.001), need for stay in NICU for more than 15 days (*P* < 0.001), presence of HMD (*P* < 0.001) and presence of NEC (*P* < 0.001) to be significant for the development of any ROP. Univariate analysis showed birth weight (*P* < 0.001), GA (*P* < 0.001), presence of IVH (*P* < 0.001), need for surfactant (*P* < 0.001), presence of bacterial infection (*P* < 0.001), presence of fungal infection (*P* < 0.001), presence of combined fungal and bacterial infection (*P* < 0.001), need for stay in NICU for more than 15 days (*P* < 0.001), presence of HMD (*P* < 0.001), presence of PDA(*P* = 0.005) and presence of NEC (*P* < 0.001) to be significant for the development of MTD. However, many of these factors were not found to be significant when subjected to more rigorous multivariate analysis. The risk factors identified by the multivariate analysis are summarized in Table 3 for any ROP and Table 4 for MTD.

**Table 3:** Multivariate analysis of risk factors for the development of acute retinopathy of prematurity-significant factors

Variable	Number (%)	Number with ROP (%)	*P* value	Odds ratio	95% CI†
Birth weight (g)					
≤800	45(7.5)	42(93.3)	<0.001	13.75	3.66-1.54
801-1000	94 (15.6)	75 (79.7)	<0.001	6.71	3.44-13.06
1001-1250	132 (22)	56 (42.4)	0.003	2.09	1.27-3.44
≥1251	328 (54.7)	60 (18.2)			
GA‡ (weeks)					
≤28	107 (17.8)	93 (86.9)	<0.001	4.02	1.85-8.75
29-31	181 (30.2)	75 (41.4)	0.05	1.6	0.99-2.59
≥32	311 (51.9)	65 (20.9)	-	-	-
Surfactant					
Given	353 (58.9)	165 (46.7)	0.03	1.72	1.04-2.83
Not given	246 (41)	68 (27.6)	-	-	-
Stay in NICU§				
<15 days	268 (44.7)	82 (30.5)	0.3	0.75	0.44-1.29
≥15 days	122 (20.3)	99 (81.1)	0.03	2.25	1.05-4.82
Nil	209 (34.8)	52 (24.8)	-	-	-

*P* value significant if < 0.05;

† Confidence interval;

‡ Gestational age;

§ NICU-Neonatal intensive care unit, blank boxes are comparing entities

**Table 4:** Multivariate analysis for risk factors for the development of modified threshold disease-significant factors

Variable	No. (%)	Babies with MTD (% in the group)	*P* value	Odds ratio	95% confidence interval (CI)†
Birth weight (g)					
≤800	45 (7.5)	20 (42.5)	0.001	22.86	3.87-134.82
801- 1000	94 (15.7)	20 (42.5)	0.009	9.63	1.76-52.55
1001-1250	132 (22.0)	5 (10.6)	0.13	3.77	0.65-21.65
≥1251	328 (54.7)	2 (4.2)	-	-	-
Surfactant					
Given	353 (58.9)	45 (12.7)	0.05	4.41	0.94-20.56
Not given	246 (41.1)	2 (0.8)	-	-	-
Bacterial sepsis					
Present	121 (20.2)	32 (26.4)	0.002	3.27	1.51-7.05
Absent	478 (79.8)	15 (3.1)	-	-	-

*P* value significant if < 0.05;

† Confidence interval; Blank boxes are comparing entities

## Discussion

The incidence of ROP in our study of 599 babies was 38.4% which is comparable to other studies.[7,12,15,16] In a study conducted in our center in 1996-97, ROP was found among 64.5% of babies with birth weight of less than 2000 g.[17] The higher incidence of ROP could be due to smaller babies in the earlier cohort.[17] A decreasing incidence of ROP has been observed by some authors[4,5,19] and this has been attributed to the introduction of use of Surfactant[5] or to the improving neonatal care and better monitoring of oxygen saturation.[19]

In our study the incidence of any ROP among babies with birth weight of less than 1251 g was 63.8%, which is comparable to 65.8% noted in the CRYO-ROP study[3] and 68% in Early Treatment of Retinopathy of Prematurity (ETROP) incidence study.[20] This observation illustrates that the incidence of ROP has remained the same as it was 20 years ago in babies with birth weight of less than 1251 g and hence efforts should be made to include all the babies with birth weight of less than 1251 g for ROP screening.

The incidence of ROP needing laser treatment in our series was 7.8% which is comparable to other reports.[8,15] Some studies have reported incidence of less than 5% of severe ROP needing laser treatment.[4–6] Chow *et al.*[21] reported zero incidence of severe ROP needing treatment and attributed it to a protocol of improved management of oxygen administration.

The ETROP incidence study[20] reported severe pre-threshold ROP in 36.9% as against 27% incidence of threshold disease in the CRYO ROP study[3] underlining the continued occurrence of this sight-threatening stage of ROP. In our study in the subgroup of 271 babies with birth weight of less than 1251 g, the incidence of MTD was 16.6%. The lower incidence in our study may be due to comparatively larger babies in our cohort. Table 5 compares the incidence of ROP and severe ROP, average birth weight and GA in various studies. Gilbert *et al*.[22] observed that in less developed countries, babies with higher birth weight may develop severe ROP as compared to more developed countries.

**Table 5:** Comparison of various studies with reported incidence of retinopathy of prematurity and severe retinopathy of prematurity needing treatment and average birth weight and gestational age

Study/year	Number	Birth weight (g)	GA	Any ROP %	Threshold %
Phan *et al*.[8]	227	1512	31	45.8	9.1
Al Essa *et al*.[17]	234	1145	30.22	64.5	14.5
Al Amro *et al*.[16]	195	1103.3	28.4	37.4	9.7
Seiberth *et al*.[11]	402	1148	29.9	36	NA
ETROP study[20]	6998	907	27.4	68	36.9	
This study	599	1270	31.1	38.8	7.8

The aim of our study included identifying the risk factors for the development of any ROP and severe ROP. We found that sex, multiple pregnancy or IUGR were not found to be significant risk factors for the development of any ROP or MTD by univariate or multivariate analysis.

Several factors were found to be significant for the development of any ROP and MTD by univariate analysis (see section on Results). Tables 3 and 4 show the factors found to be significant by more rigorous multivariate analysis for the development of any ROP and MTD respectively.

Low birth weight,[6,7,10–12] low GA[5,10–12] surfactant,[6] and need for longer stay in the hospital,[10] bacterial sepsis[7,10] and presence of IVH[7] have been reported to be significant risk factors for development of any ROP in the literature. Lower GA at birth indicates larger area of avascular retina which predisposes the baby to developing ROP.

Multivariate analysis found low birth weight, bacterial sepsis and surfactant to be significant for the development of MTD [Table 4]. Low birth weight has been consistently found to be significant for the development of threshold disease in other studies too.[6,12,17] Some studies have found presence of IVH[13] and fungal infection[14] to be significant for the development of threshold disease.

The outcome of treated babies was satisfactory in our study with unfavorable outcome seen in 6.38% of the treated babies. The CRYO- ROP[3] and ETROP[23] studies reported unfavorable outcome in 21.8% and 11% of treated babies respectively. The incidence of Zone I disease (8.13%) in our study is also comparable to that in the ETROP study (9.1%).[23] It is interesting to observe that the average clock hours of extraretinal proliferations among the treated babies was 9.6 clock h in the CRYO- ROP study.[3] Our decision to treat babies at MTD rather than at the stage of classic threshold disease recommended by CRYO- ROP study was based on the observation by Fleming *et al*.[24] who noted more favorable outcome when the disease was treated with less circumferential involvement of Stage 3. The natural history study of CRYO- ROP[25] showed that babies with greater extent of Stage 3 + disease had greater probability of developing unfavorable outcome. As we have noted in the section on results, only one out of 70 eyes treated at MTD level had unfavorable outcome as against four out of 16 eyes treated at classical threshold or higher. Azad *et al*.[26] in a randomized study observed that early treatment at pre-threshold stage had less incidence of unfavorable outcome than those treated at classical threshold disease. Our study shows that, setting a lower extent of circumferential extent of Stage 3 disease as the target for treatment, allows clinicians to screen more often and treat progressive form of ROP in time. We would like to acknowledge that this study was conducted before the results of the ETROP study[23] were published. Diagnosis of Type I disease as defined in the ETROP study[23] depends upon the recognition of plus disease. Chang *et al*.[27] showed that inter-expert agreement of diagnosis of plus disease was imperfect. We feel that when the diagnosis of plus disease is equivocal in a case of ROP with Stage 3, laser therapy at the stage of MTD may be an alternative approach in taking decision to treat ROP.

## Conclusion

The incidence of both any ROP and ROP needing treatment in our study was comparable to other studies. Birth weight is the most important risk factor for both any ROP and severe ROP. Treating babies at the stage of MTD has yielded low incidence of unfavorable outcome in our study.
